# Supplementary material for: An Improved HRPE-Based Transcriptional Output Reporter to Detect Hypoxia and Anoxia in Plant Tissue
Source: Biosensors (Basel). 2020 Dec 3;10(12):197. doi: 10.3390/bios10120197 (PMC7761731; doi:10.3390/bios10120197)
Supplement: Supplementary file 1 [file biosensors-10-00197-s001.zip › biosensors-1002280-suppl/Supplementary Figure 1.pdf]

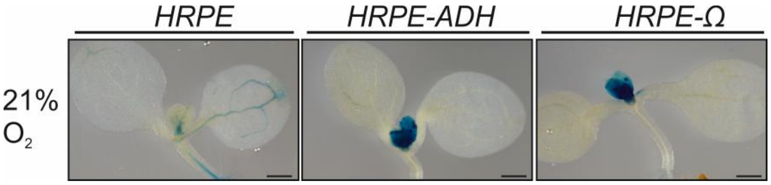

Supplementary Figure 1. Overnight histochemical GUS staining of *HRPE:GG* variants grown at 21% O_2. Scale bar: 1 mm.
