# Supplementary material for: An Improved HRPE-Based Transcriptional Output Reporter to Detect Hypoxia and Anoxia in Plant Tissue
Source: Biosensors (Basel). 2020 Dec 3;10(12):197. doi: 10.3390/bios10120197 (PMC7761731; doi:10.3390/bios10120197)
Supplement: Supplementary file 1 [file biosensors-10-00197-s001.zip › biosensors-1002280-suppl/Supplementary tables.pdf]

Supplementary table 1. HRPE promoters sequences used and generated in this study.

Name	Sequence
HRPE	TGAATCAGGGTGAAGAAAAACAGCGGCGGTTCTTGAATCAGGGTGAAGAAAAACA<br>GCGGCGGTTCTTGAATCAGGGTGAAGAAAAACAGCGGCGGTTCTTGAATCAGGGTG<br>AAGAAAAACAGCGGCGGTTCTTGAATCAGGGTGAAGAAAAACAGCGGCGGTTCTCG<br>CAAGACCCTTCCTCTATATAAGGAAGTTCATTTCATTTGGAGAGGTATTTTTACAACA<br>ATTACCAACAACAACAAACAACAACTAGT
HRPE-Ω	TGAATCAGGGTGAAGAAAAACAGCGGCGGTTCTTGAATCAGGGTGAAGAAAAACA<br>GCGGCGGTTCTTGAATCAGGGTGAAGAAAAACAGCGGCGGTTCTTGAATCAGGGTG<br>AAGAAAAACAGCGGCGGTTCTTGAATCAGGGTGAAGAAAAACAGCGGCGGTTCTCG<br>CAAGACCCTTCCTCTATATAAGGAAGTTCATTTCATTTGGAGAGGTATTTTTACAACA<br>ATTACCAACAACAACAAACAACA**CTCCGGTATTTTTACAACAATACCACAACAAA**<br>**ACAAACAACAAACAACATTACAATTTACTATTCTAGTCGA**
HRPE-ADH	TGAATCAGGGTGAAGAAAAACAGCGGCGGTTCTTGAATCAGGGTGAAGAAAAACA<br>GCGGCGGTTCTTGAATCAGGGTGAAGAAAAACAGCGGCGGTTCTTGAATCAGGGTG<br>AAGAAAAACAGCGGCGGTTCTTGAATCAGGGTGAAGAAAAACAGCGGCGGTTCTCG<br>CAAGACCCTTCCTCTATATAAGGAAGTTCATTTCATTTGGAGAGGTATTTTTACAACA<br>ATTACCAACAACAACAAACAACA**ACGCCAAGTGGAAAGAGCGTTCGAGAGAACA**<br>**AGGCAAAACCAAATACGCCCCTAGTATTCTACAGATGTCGACTGGATAATTACAA**<br>**AAGATTTCAATAAACAGTACTAATTAATTTCTAGTGGTGAGTTTTTGTAAATATCT**<br>**ACTTCTTCCAATTACCAGCTGCTATATAAATCCCCTTCTCTGTTTCTCTTTTCTTAC**<br>**ATCACAATCACACAAAACTAACAAAAGATCAAAAGCAAGTTCTTCACTGTTGATA**

Supplementary table 2. List of nucleotide sequences generated in this study

Name	Sequence
UnaG	CACC**ATG**GTCGAGAAGTTCGTTGGAACCTGGAAGATCGCTGACAGCCATAACTTCG<br>GAGAGTACCTCAAGGCTATCGGGGCTCCTAAAGAACTCTCTGATGGTGGTGATGCTA<br>CTACCCCGACTCTCTACATCTCTCAAAAGGACGGTGACAAGATGACCGTGAAGATCG<br>AAAACGGACCTCCTACCTTCCTCGATACCCAGGTTAAGTTCAAGCTCGGGGAAGAGT<br>TCGATGAGTTCCCATCTGATAGACGTAAGGGCGTGAAGTCTGTGGTTAACCTCGTTG<br>GAGAGAAGCTCGTGTACGTTCAAAAGTGGGACGGAAAGAGACTACCTACGTCAGA<br>GAGATCAAGGACGGAAAGCTCGTGGTGACTCTCACTATGGGAGATGTTGTGGCTGTG<br>AGGTCTTACAGACGTGCTACTGAATGA
iLov	CACC**ATG**ATCGAGAAGAACTTCGTGATTACCGATCCTAGGCTCCCTGACAACCCTAT<br>CATCTTCGCTTCAGATGGCTTCCTCGAGCTGACCGAATACTCTAGAGAAGAGATCCT<br>CGGTAGGAACGCTAGATTCCTTCAAGGACCTGAGACTGATCAGGCCACCGTGCAAA<br>AGATCAGAGATGCTATCAGGGACCAGCGTGAGACTACTGTTCAGCTCATCAACTACA<br>CCAAGAGCGGCAAGAGATTCTGGAACCTTCTTCATCTCCAGCCTGTGAGGGATCAGA<br>AGGGTGAGCTTCAATACTTCATCGGAGTGCAGCTCGATGGATCTGATCACGTTTGA
Pp2FbFP	CACC**ATG**ATCAACGCTAAGCTCCTCCAGCTCATGGTCGAGCATTCTAACGATGGAAT<br>CGTGGTGGCTGAGCAAGAGGGAAACGAGAGCATCATGATCTACGTGAACCCTGCTT<br>TCGAGAGGCTCACTGGATACTGCGCTGATGATATCCTCTACCAGGATGCTAGATTCCT

	CCAAGGTGAGGATCACGATCAACCGGGAATCGCTATTATCCGTGAGGCTATCAGAG<br>AGGGTAGACCTTGTTGTCAGGTGCTCCGTAACTACCGTAAGGACGGATCTTTGTTCTG<br>GAACGAGCTTAGCATCACCCCTGTGCATAACGAGGCTGATCAGTTGACCTACTACAT<br>CGGGATCCAGAGAGATGTGACTGCTCAGGTTTTCGCTGAAGAGAGGGTTAGAGAGC<br>TTGAGGCTGAAGTTGCTGAGCTTAGAAGGCAACAGGGACAAGCTAAGCACTGA
